# Machbarkeit und Akzeptanz videobasierter Physiotherapie: Neues Versorgungsangebot für ältere Menschen während der COVID-19-Pandemie

**DOI:** 10.1007/s00391-021-01899-3

**Published:** 2021-04-30

**Authors:** Lisa Happe, Sandra Lau, Jessica Koschate, Rebecca Diekmann, Andreas Hein, Tania Zieschang

**Affiliations:** 1 Abteilung für Assistenzsysteme und Medizintechnik, Department für Versorgungsforschung, Carl von Ossietzky Universität Oldenburg, Ammerländer Heerstr. 140, 26129 Oldenburg, Deutschland; 2 Abteilung für Geriatrie, Department für Versorgungsforschung, Carl von Ossietzky Universität Oldenburg, Ammerländer Heerstr. 140, 26129 Oldenburg, Deutschland

**Keywords:** Körperliche Aktivität, Videotherapie, Teletherapie, SARS-CoV-2, Ältere Patienten, Physical activity, Video therapy, Teletherapy, SARS-CoV-2, Older patients

## Abstract

**Hintergrund und Ziel:**

Einschränkungen des öffentlichen Lebens durch die COVID-19-Pandemie dienen insbesondere dazu, Risikogruppen vor einer Ansteckung zu schützen. Darunter fallen auch ältere, multimorbide Patienten, für die körperliche Inaktivität und Auslassen von Maßnahmen wie Physiotherapie jedoch negative Folgen haben können. Die vorliegende Studie untersucht die Machbarkeit und die subjektive Bewertung videobasierter Physiotherapie (VT).

**Methoden:**

Von April bis Juni 2020 nahmen 4 Einrichtungen mit 9 Patienten (6 Frauen, 64 bis 82 Jahre) an der Studie teil, die mit Tablets ausgestattet wurden. Durch semistrukturierte Telefoninterviews wurden körperliche Aktivität, funktionelle Kompetenz und Partizipation vor und während den Einschränkungen bei 8 Patienten erfasst. Patienten und Therapeuten wurden zu ihren subjektiven Erfahrungen mit der VT befragt.

**Ergebnisse:**

Es fanden insgesamt 92 VT-Einheiten statt. Die Umsetzung der Übungen wurde als gut bis sehr gut bewertet. Insgesamt zeigte sich eine hohe Akzeptanz der VT. Vier von 8 Patienten beschrieben eine subjektive Reduzierung ihrer körperlichen Aktivitäten aufgrund der Einschränkungen. Diese Veränderungen wurden über die verwendeten Fragebogen zur Partizipation und zur körperlichen Aktivität nicht abgebildet.

**Diskussion:**

Bei älteren Patienten ist VT mit geringer technischer Unterstützung machbar. Sowohl in Pandemiesituationen als auch in anderen Kontexten stellt sich VT als eine realisierbare Ergänzung oder Alternative zur normalen Physiotherapie dar. Weitere Studien zur Identifikation von geeigneten Patientengruppen, Effektivität der VT und Weiterentwicklung inhaltlicher Aspekte sind dringend notwendig.

**Zusatzmaterial online:**

Zusätzliche Informationen sind in der Online-Version dieses Artikels (10.1007/s00391-021-01899-3) enthalten.

## Abstract (en)

**Background and objective:**

Restrictions in public life during the COVID-19 pandemic mainly addressed the safety of high-risk older multimorbid patients to protect them from infection. Nevertheless, detrimental aspects of the lockdown for older people are a reduction in physical activity and limited access to physiotherapy, which are likely to have a negative impact on the health status. This study examined the feasibility of video-based physiotherapy (VT) and the subjective rating of VT by patients and therapists.

**Methods:**

From April to June 2020, 4 facilities with 9 patients (6 women, 64–82 years old) participated in the study and were provided with technical equipment. Semistructured telephone interviews were conducted in eight patients to assess physical activity, functional ability and participation before and during the restrictions. Both patients and therapists were asked about their subjective experiences with VT.

**Results:**

A total of 92 VT sessions took place. Performance of the exercises and comprehension of instructions were rated as good to very good. The level of acceptance was high. Of the patients four described a perceived reduction in their physical activities due to the public restrictions; however, in the questionnaires no substantial differences in participation and physical activity were found.

**Discussion:**

The use of VT is feasible in older patients. Only minor technical support is needed. In pandemic situations or in other contexts VT appears to be a promising supplement or alternative to normal physiotherapy. Further studies are needed to identify suitable patient groups, to prove efficacy and to develop further content-related aspects of VT.

## Hintergrund

In Deutschland kam es im Verlauf der COVID-19-Pandemie zwischen April und Juni 2020 zu Einschränkungen des öffentlichen Lebens, die Risikogruppen für einen schweren COVID-19-Verlauf wie ältere, multimorbide Personen schützen sollen. Eine Verkleinerung des täglichen Bewegungsradius und die damit einhergehende reduzierte körperliche Aktivität haben insbesondere bei älteren Menschen weitreichende negative Auswirkungen auf Selbstständigkeit und gesellschaftliche Partizipation. Physiotherapie als eine Maßnahme zur Aktivitätsförderung und zum Erhalt der Muskelkraft ist daher höchst relevant. Es ist zu erwarten, dass ein Aussetzen von Physiotherapieterminen aus Sorge vor einer Ansteckung negative gesundheitliche Folgen nach sich zieht. Bei älteren Menschen führt im Vergleich zu Jüngeren eine Reduktion der körperlichen Aktivität schneller zu einem substanziellen Abbau von Muskelmasse [13] und Ausdauerleistungsfähigkeit [12]. Daraus resultierend droht ein erhöhtes Sturzrisiko [1].

Basierend auf dem Beschluss des Gemeinsamen Bundesausschuss zu Sonderregelungen bei der Heilmittelverordnung aufgrund der COVID-19-Pandemie bestand bis zum 30.06.2020 die Möglichkeit, Physiotherapie als Videobehandlung (VT) durchzuführen [9].

Für den deutschen Versorgungskontext stellt die VT eine neue Therapieoption dar. Der Weltverband für Physiotherapie veröffentlichte bereits 2019 einen Bericht zu digitalen Therapiemethoden mit dem Hinweis, dass eine gute Versorgungsqualität und die Nutzungsbereitschaft des Patienten vorausgesetzt werden müssen [16]. International wurden bereits einzelne Studien zur VT durchgeführt. Zur Behandlung von Patienten mit muskuloskelettalen Beschwerden konnten positive Effekte auf Schmerz- und Funktionsparameter festgestellt werden [2]. Bei Patienten mit COPD [6] und mit chronischer Herzinsuffizienz [8] zeigen die Ergebnisse, dass ein in Echtzeit angeleitetes Online-Gruppentraining genauso effektiv war wie die Teilnahme an einem „Vor-Ort“-Gruppen-Training. Für Einzelbehandlungen älterer Menschen mittels VT liegt bislang keine Evidenz vor. In dieser Gruppe können technische Vorkenntnisse und Ausstattung für die Nutzung von Videosystemen nicht vorausgesetzt werden. So hatten 2017 zwar 89 % der 61- bis 66-Jährigen in Deutschland Zugang zum Internet, jedoch nur 39 % der 79- bis 84-Jährigen [7]. Rückschlüsse zur Technikkompetenz sind daraus nicht direkt ableitbar. Um Teilnehmern unabhängig von ihrer Technikerfahrung den Zugang zur VT zu ermöglichen, wird die VT implementiert und eine umfassende Unterstützung angeboten.

## Fragestellung

Im Rahmen dieser Studie soll untersucht werden:

1. ob und mit welchem Unterstützungsbedarf eine VT mit Personen ab 60 Jahren durchführbar ist,
2. wie die neue Form der Therapie subjektiv von Physiotherapeuten und Patienten wahrgenommen wird, und
3. inwieweit die pandemiebedingten Einschränkungen des öffentlichen Lebens Auswirkungen auf die Partizipation und die körperliche Aktivität der Patienten haben.

## Methode

### Studienpopulation

In diese einarmige Machbarkeitsstudie wurden Patienten ab 60 Jahren im Raum Oldenburg und den angrenzenden Gemeinden eingeschlossen, die eine gültige Verordnung für ein physiotherapeutisches Heilmittel hatten, das als VT umgesetzt werden konnte (aktive Bewegungs- und Atemtherapie). Die Rekrutierung fand von Mitte April 2020 bis Mitte Juni 2020 statt, da die Abrechnungsmöglichkeit der Verordnung einer VT zum 30.06.2020 vorerst endete. Die Ansprache der potenziellen Probanden erfolgte primär über Physiotherapiepraxen. Die Aufklärung über die Studie sowie die Einwilligung erfolgten telefonisch.

### Intervention

Die Patienten erhielten vorkonfigurierte 10-Zoll-Tablets (Lenovo YT-X705L, Lenovo Group Limited, Quarry Bay, Honkong, China) mit Mobilfunkanbindung und einer konventionellen Videotelefonie-Software (TrueConf, Fa. TrueConf LLC., Moskau, Russland), um einen visuellen und verbalen Austausch zwischen Patient und Physiotherapeut zu ermöglichen. Zudem wurde eine detaillierte, mit Fotografien veranschaulichte, gedruckte Anleitung zu Inbetriebnahme und Nutzung des Geräts ausgehändigt. Die Praxen erhielten bei Bedarf ebenfalls adäquates technisches Equipment, eine Einweisung und Support. Die inhaltliche Gestaltung der VT-Einheit wurde vom Studienteam nicht vorgegeben, beeinflusst oder bewertet.

### Messinstrumente

Die Therapeuten beantworteten nach jeder Therapieeinheit Fragen zu Verbindungsqualität und Durchführbarkeit der Behandlung auf einem Bewertungsbogen mit einer 5‑stufigen Likert-Skala. Es wurde zudem abgefragt, welche Körperregionen behandelt wurden und, zum Abschluss der Studienteilnahme, welche Behandlungsinhalte (z. B. Atemtherapie, Kräftigungsübungen) mit welchen Hilfsmitteln am Patienten durchgeführt wurden. Ergänzend dazu wurden sowohl die Therapeuten als auch Patienten in einem semistrukturierten Interview zu ihrer subjektiven Einschätzung der VT befragt (Zusatzmaterial online: 1, 2).

Zur Erfassung der sozioökonomischen und gesundheitsbezogenen Daten sowie der körperlichen Aktivität und Partizipation erfolgten standardisierte, semistrukturierte Telefoninterviews mit den Patienten bei Eintritt in die Studie und nach 6 Wochen (t2). Ein Interview dauerte zwischen 45 und 60 min und orientierte sich an einem Interviewleitfaden. Das erste Telefoninterview enthielt sowohl eine retrospektive Erhebung (t0) vor den COVID-19-bedingten Einschränkungen als auch eine Datenerhebung zur aktuellen Situation (t1). Die Partizipation im Sinne funktioneller Alltagskompetenz als relevantes Merkmal zum Erhalt der Selbstständigkeit von zu Hause lebenden älteren Menschen wurde mittels LUCAS-Funktionsindex eingestuft. Die Zuordnung in die Kategorien frail, pre-frail, post-robust und robust erfolgte anhand von 12 Fragen zu Alltagsverhalten und Aktivitäten, Risiken eines funktionellen Abbaus, körperlichen Reserven sowie sozialen Schutzfaktoren [3]. Zur Abschätzung der körperlichen Aktivität wurden individuelle Freizeitaktivitäten anhand einer adaptierten Version des Minnesota Leisure Time Physical Activity Questionnaire (MLTPAQ) ermittelt (Zusatzmaterial online: 3). Die Auswahl der 14 Items wurde in Anlehnung an die Verwendung des MLTPAQ beim Frailty-Phänotyp nach Fried et al. [4] getroffen und durch regionale und altersentsprechende Angebote angepasst, um unterschiedliche Intensitäten abzubilden. Aus den individuellen Angaben wurde der Energieverbrauch (kcal) pro Woche berechnet [4, 14].

### Datenauswertung

Die Interviewbögen wurden von zwei Studienassistentinnen unabhängig voneinander in die Studien-Software REDCap (Version 10.6, Vanderbilt University, Nashville, TN, US) übertragen und von einer dritten Person überprüft. Die demografischen Angaben und Gesundheitsdaten wurden deskriptiv mit der Software SPSS (Version 26, SPSS Inc., Chicago, IL, US) und die subjektive Einschätzung zur VT mit MAXQDA (Version 20, VERBI Software GmbH, Berlin, Deutschland) ausgewertet. Die qualitativen Informationen aus den offenen Fragen des Telefoninterviews zur subjektiven Einschätzung der VT wurden inhaltsanalytisch nach Kuckartz durch zwei Untersucherinnen unabhängig voneinander ausgewertet [10]. Dazu wurden a priori Hauptkategorien anhand der Leitfragen gebildet. Während des Kodierens wurden induktiv Unterkategorien aus dem Textmaterial gebildet. Die Kategorien und kodierten Textstellen wurden anschließend von den Untersucherinnen verglichen, diskutiert und konsentiert.

## Ergebnisse

**Figure 1:**
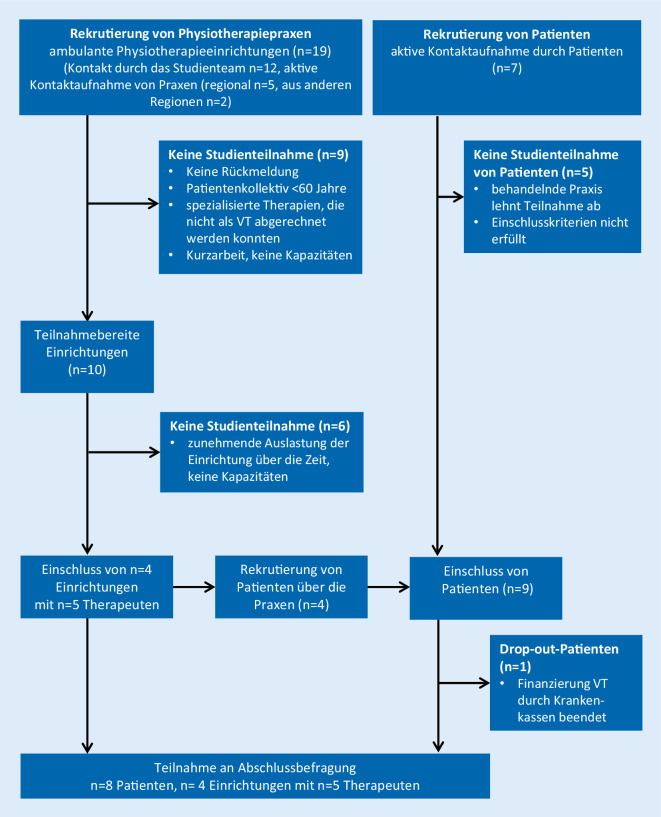

Im Zeitraum vom 27.04.2020 bis 10.06.2020 wurden insgesamt 19 physiotherapeutische Einrichtungen im Raum Oldenburg und dem Umland kontaktiert. Zwei Anfragen von Praxen aus entfernteren Regionen konnten aufgrund der personellen Ressourcen nicht berücksichtigt werden. Sieben Patienten meldeten sich direkt beim Studienteam, von denen 2 Personen eingeschlossen wurden. Insgesamt wurden 4 Einrichtungen mit 9 Patienten und 5 Therapeuten in die Studie eingeschlossen (Details: Abb. 1).

Das Durchschnittsalter der eingeschlossenen Patienten (6 Frauen, 3 Männer) lag bei 72,8 (± 5,5) Jahren. Die Patientencharakteristika sowie die individuellen Ergebnisse des LUCAS-Fragebogens [5] und des Kalorienverbrauchs über Freizeitaktivitäten nach MLTPAQ [14] sind als online Material verfügbar (Zusatzmaterial online: 4). Bezüglich der erhobenen persönlichen Angaben und Instrumente ergaben sich keine eindeutigen Unterschiede. Hinsichtlich der Aktivität zeigten die erhobenen Daten zu Partizipation und Mobilität, dass dieses Kollektiv eher individuelle Übungsprogramme durchführt (*n* = 7). Einen direkten Zusammenhang zwischen der Reduzierung von körperlichen Aktivitäten und der COVID-19-Pandemie berichteten 4 Teilnehmer.

Im Studienzeitraum fanden insgesamt 92 VT-Einheiten statt. Die Physiotherapeuten führten zwischen 6 und 49 Therapieeinheiten durch. Bei den Patienten variierte die Anzahl der durchgeführten Therapien zwischen 2 und 20, mit einer Behandlungsdauer von mindestens 20 min. Alle Therapeuten gaben an, allgemeine Bewegungs- und Kräftigungsübungen während der VT durchgeführt zu haben. Vier Therapeuten setzten zudem Hockergymnastik und Balance-Übungen ein. Atemtherapie wurde von 2 Therapeuten angewandt. Zum Einsatz kamen verschiedene Hilfsmittel (wie Fitnessband und Wasserflasche).

Im Unterschied zu den Patienten erhielten die Therapeuten eine etwas umfangreichere Einweisung von 30 min in die Funktionen der Software, um bei kleineren technischen Problemen agieren zu können. Im Verlauf der Studie gab es bei 6 Patienten und einem Therapeuten weiteren Unterstützungsbedarf. Bei 4 Teilnehmern traten im Studienzeitraum Verbindungsstörungen auf.

**Figure 2:**
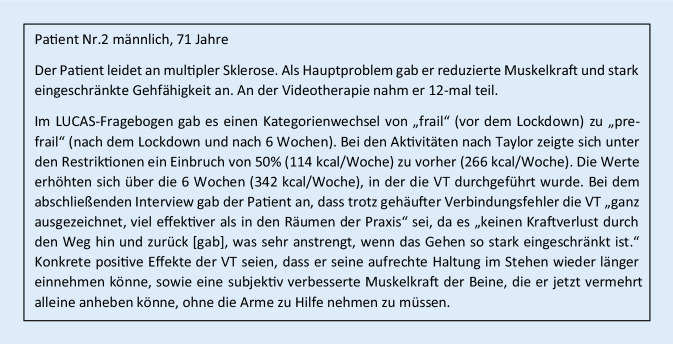

Die von den Patienten subjektiv wahrgenommenen Erfahrungen und Effekte der VT waren heterogen. Exemplarisch zeigen Abb. 2 und 3 die individuellen Rückmeldungen von 2 Patienten.

**Figure 3:**
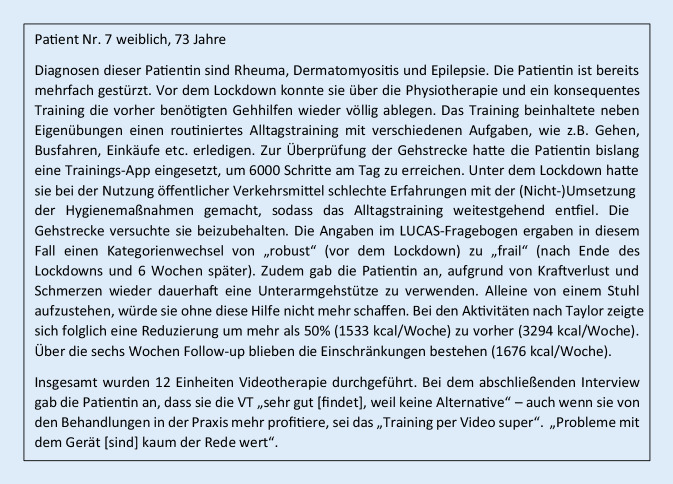

**Figure 4:**
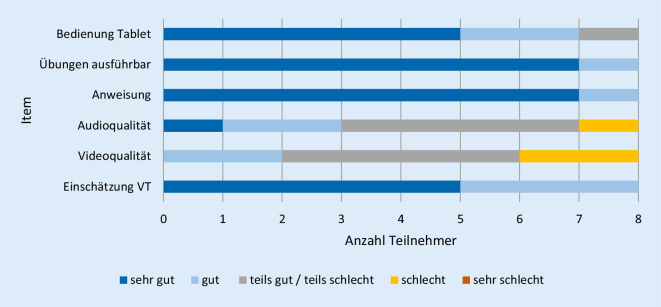

Aus Perspektive der Patienten wurde die Audio- und Videoqualität als mäßig bis schlecht bewertet. Alle anderen Items, v. a. die Umsetzung der Übungen und das Befolgen von Anweisungen, wurden als sehr gut bis gut eingeschätzt (Abb. 4).

Der einzige Therapieabbruch lag an der zeitlich limitierten Abrechnungsmöglichkeit für VT. Sicherheitsbedenken wurden von den Patienten nicht geäußert. Insgesamt gaben die Patienten an, sich mit VT mehr bewegt zu haben als ohne, und dass sie sich auch zukünftig die VT als eine Alternative zur herkömmlichen PT vorstellen können. Die Analyse der Patientenkommentare, bezogen auf die Gesamteinschätzung, ergab drei inhaltliche Schwerpunkte: technikassoziierte Kritik, Vor- und Nachteile der VT und perspektivischer Nutzen. Technikassoziierte Kritik beinhaltete hauptsächlich Probleme mit der Internetverbindung, die zu Bild- und Tonproblemen führte, aber auch eine eingeschränkte Bildperspektive wurde bemängelt. Als Vorteile wurde v. a. die Weiterführung der Therapie genannt. Nachteilig wurden die eingeschränkte Haltungskorrektur und fehlende Therapiegeräte empfunden. Perspektivisch gaben die Patienten Impulse zur Weiterentwicklung der VT wie z. B. ein hinterlegtes Video-Übungsprogramm oder die Aufzeichnung der Therapie.

**Figure 5:**
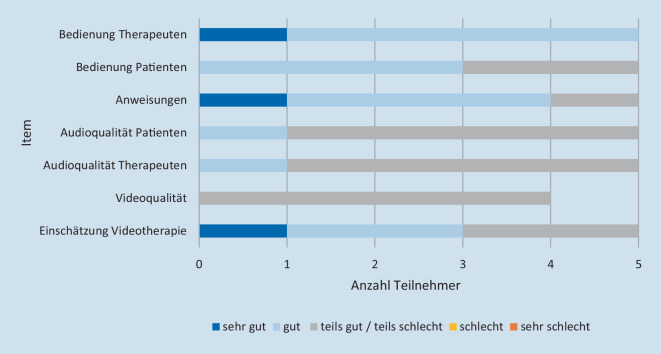

Aus Therapeutensicht wurde die eigene Bedienung des Tablets besser bewertet als die der Patienten. Die Audio- und Videoqualität wurde ebenfalls als eher mäßig beurteilt. Bei der Einschätzung zur VT allgemein und bei der Umsetzung der Anweisungen zeigte sich ein ambivalentes Stimmungsbild (Abb. 5).

Insgesamt beurteilten 4 Physiotherapeuten die Aufnahme von VT in den Leistungskatalog der Krankenkassen als sinnvollen Schritt. Ein Physiotherapeut gab Sicherheitsbedenken an, da z. B. bei einem Sturz nicht eingegriffen werden könnte. Weiterer Unterstützungsbedarf zur Umsetzung von VT wurde von den Physiotherapeuten bei sich selbst (*n* = 3), den Kollegen und Mitarbeitern oder in Praxen (*n* = 3) benannt. Die Erfahrungen der Therapeuten zu den Vor- und Nachteilen der VT sind als online Material verfügbar (Zusatzmaterial online: 5).

## Diskussion

Im Rahmen dieser Studie wurde untersucht, ob und mit welchem Unterstützungsbedarf eine VT bei Personen ab 60 Jahren möglich ist, und wie die neue Therapieform von Physiotherapeuten und Patienten bewertet wird. Die Anzahl der durchgeführten Therapien sowie die insgesamt positive Bewertung durch Patienten und Therapeuten zeigen, dass die VT als Behandlungsalternative und/oder -ergänzung zur herkömmlichen Physiotherapie grundsätzlich erstrebenswert ist. Die Ergebnisse der Studie geben Hinweise darauf, dass eine VT sicher ist und keinen nachteiligen Effekt erzeugt. Die Hälfte der Patienten beschrieb negative Auswirkungen der pandemiebedingten Einschränkungen auf ihre körperliche Leistungsfähigkeit. Die Effektivität der VT anhand geeigneter Assessments oder ein Vergleich mit konventioneller Therapie wurden in dieser Studie nicht untersucht. Diese erste Studie zeigt jedoch, dass VT im Kollektiv älterer, gebrechlicher Patienten umsetzbar ist und von Patienten und Therapeuten angenommen wird.

In den Praxen zeigte sich im Umgang mit der VT ein ähnlicher Unterstützungsbedarf wie bei den Patienten. Keine der teilnehmenden Praxen hatte Erfahrung mit Videotelefonie. Im Praxisalltag kann nach unseren Erkenntnissen ein routinierter Umgang mit einem Computer, dem Internet und Software nicht als gegeben angenommen werden. Eine Befragung australischer Physiotherapeuten zeigte einen signifikanten Zusammenhang zwischen einem geringen Interesse der Therapeuten an der Durchführung einer VT und der eigenen Unsicherheit bei der Nutzung von Video-Software sowie der Unerfahrenheit hinsichtlich Telerehabilitation [11]. Schulungen der Physiotherapeuten und Behandlungsleitlinien sollten für die Implementierung der VT in den Versorgungsalltag daher unbedingt eingeplant werden. Schwierigkeiten bei der Verbindungsqualität traten insbesondere im ländlichen Bereich auf.

In dieser Studie, wie in den wenigen Publikationen zur subjektiven Bewertung dieser Therapieform, wurde VT als neue Therapiemethode von Patienten und Therapeuten positiv bewertet. Bei einer Umfrage sahen Physiotherapeuten die Vorteile einer VT v. a. in der Zeitersparnis für den Patienten, und dass die Therapie für Patienten von zu Hause durchgeführt werden kann [11]. Ein Review über die Effektivität von VT gegenüber konventioneller Physiotherapie bei älteren Menschen zeigte keine signifikanten Unterschiede zwischen den beiden Therapieformen und gab damit Hinweise auf eine Nichtunterlegenheit der VT gegenüber konventioneller Physiotherapie [15]. In einer Kohorte von Patienten mit COPD konnten zudem signifikant geringere Drop-out-Raten bei einer gruppenbasierten VT im Vergleich zur konventionellen gruppenbasierten Physiotherapie beobachtet werden [6].

Die Hälfte der Patienten gab einen negativen Einfluss der pandemiebedingten Einschränkungen auf Partizipation und körperliche Aktivität an. Aus Sorge vor einer Ansteckung blieben diese Personen vermehrt zu Hause und schränkten die Teilnahme am öffentlichen Leben ein.

In unserem Patientenkollektiv wurde deutlich, dass die körperlichen Aktivitäten über herkömmliche Instrumente nicht zufriedenstellend erfasst wurden. Die Patienten nutzten zumeist individuelle therapeutische Eigenübungsprogramme anstelle von klassischen Sport- und Bewegungsangeboten.

Für eine Übernahme in die Regelversorgung bedarf es der Identifikation geeigneter Patientengruppen sowie der Entwicklung entsprechender Therapieprotokolle.

### Limitationen

Durch die zeitliche Begrenzung der Abrechnungsmöglichkeit für VT konnten nur wenige Praxen und Patienten eingeschlossen werden, sodass die Stichprobe nicht repräsentativ ist. Erschwerend für eine erfolgreiche Rekrutierung war, dass sowohl Patient als auch behandelnder Therapeut die Bereitschaft mitbringen müssen, an einer VT teilzunehmen. Die potenzielle Bereitschaft bei Patienten und Therapeuten im Fall einer regulären Übernahme in den Katalog können wir nicht einschätzen. Die retrospektive Erhebung zur körperlichen Aktivität vor der COVID-19-Pandemie hat eine eingeschränkte Aussagekraft. Im Rahmen dieser Studie wurden keine funktionellen Assessments mit den Patienten durchgeführt, sodass keine objektiven Rückschlüsse auf die Effektivität der VT möglich sind.

### Ausblick

Die VT ist für das deutsche Versorgungssystem eine neue Behandlungsform, die in anderen Ländern etabliert ist. Aufgrund der unterschiedlichen Gesundheitssysteme und geografischen Rahmenbedingungen kann nicht von einer generellen Übertragbarkeit ausgegangen werden. Die weitere wissenschaftliche Untersuchung der VT, wie sie nach Initiierung der Berufsverbände aktuell vom gemeinsamen Bundesausschuss angestoßen wird, ist daher notwendig.

Zukünftige Studien sollten untersuchen, welche Patientengruppen sich besonders für VT eignen. Für Patienten mit erheblichen Mobilitätseinschränkungen könnte eine kombinierte Therapieform von VT und konventioneller Physiotherapie sinnvoll sein. Für die diagnostische Bewertung der Patienten gibt es für einige Untersuchungsmethoden, wie Bewegungsausmaße oder den „Timed-up-and-go“-Test, bereits Hinweise auf eine gute Reliabilität und Validität als Tele-Assessment [5]. VT-Interventionen müssen entwickelt und auf Sicherheit und Effektivität untersucht werden. Abgesehen vom Einsparen von Wegezeiten ermöglicht VT die Individualisierung von Übungen in der Häuslichkeit.

## Fazit für die Praxis

- Die VT ist eine innovative Therapiemethode, die auch bei älteren Patienten machbar ist und eine hohe Akzeptanz aufweist. Der technische Unterstützungsbedarf ist gering.
- Insbesondere in der Pandemiesituation sollte die Abrechenbarkeit durch die Kostenträger verlässlich für eine längere Dauer gewährleistet werden. Es ist notwendig, den Einsatz von VT ergänzend oder alternativ zur konventionellen Therapie auch in anderen Kontexten (bestimmte Patientengruppen, strukturarme Regionen) zu prüfen.
- Weitere Studien sollten Effektivität und Nichtunterlegenheit zur konventionellen Therapie untersuchen sowie die innovative technische Weiterentwicklung adressieren.

## Supplementary Information










