# Supplementary material for: Feasibility and acceptance of video-based physiotherapy: New medical care provision for older people during the COVID-19 pandemic
Source: Z Gerontol Geriatr. 2021 Apr 30;54(4):346–52. [Article in German] doi: 10.1007/s00391-021-01899-3 (PMC8090529; doi:10.1007/s00391-021-01899-3)
Supplement: Supplementary file 1 [file 391_2021_1899_MOESM1_ESM.pdf]

**Körperregion, die behandelt wurde** (bitte ankreuzen)	Ganzer Körper<br>Oberkörper inkl. Schulter/Arme/HWS<br>Untere Extremitäten, Hüfte, LWS
**Diagnosegruppe** (bitte ankreuzen)	Neurologie<br>Orthopädie<br>Pneumologie<br>Kardiologie<br>Sonstiges:

	**1 (sehr gut)**	**2 (gut)**	**3 (teils gut/teils schlecht)**	**4 (schlecht)**	**5 (sehr schlecht)**
Wie fanden Sie die Videotherapie insgesamt?					
Wie war die Video-Qualität?					
Wie konnten Sie ihren Therapeuten verstehen?					
Konnten Sie den Anweisungen des Therapeuten folgen?					
Konnten Sie die geforderten Übungen ausführen?					
Wie war die Bedienung des Tablets?					
Wie war die Sicherheit während der Physiotherapie?					

	Ja	Nein
Würden Sie Videotherapie als Alternative während der Corona-Krise annehmen?		
Ist die Videotherapie für Sie zukünftig eine Alternative zur normalen Physiotherapie?		
Haben Sie sich durch die Video-Therapie mehr bewegt als ohne?		
Haben Sie die Therapie vorzeitig abgebrochen?		

Bei Therapieabbruch: welcher Grund hat dazu geführt?

Zusätzliche Kommentare:
