# Supplementary material for: Feasibility and acceptance of video-based physiotherapy: New medical care provision for older people during the COVID-19 pandemic
Source: Z Gerontol Geriatr. 2021 Apr 30;54(4):346–52. [Article in German] doi: 10.1007/s00391-021-01899-3 (PMC8090529; doi:10.1007/s00391-021-01899-3)
Supplement: Supplementary file 2 [file 391_2021_1899_MOESM2_ESM.pdf]

**Datum**	
**Probanden Alter**	
**Probanden Geschlecht**	
**Diagnose**	
**Behandelte Körperregion** (bitte ankreuzen)	Ganzer Körper<br>Oberkörper, inkl. Schulter, Arme, HWS<br>Untere Extremität inkl. Hüfte, LWS

	**1 (sehr gut)**	**2 (gut)**	**3 (teils gut/teils schlecht)**	**4 (schlecht)**	**5 (sehr schlecht)**
Wie fanden Sie die Videotherapie?					
Wie war die Video-Qualität?					
Wie konnten Sie ihren Probanden verstehen?					
Konnte der Proband Ihren Anweisungen folgen?					
Wie bewerten Sie die Sicherheit des Probanden während der Therapie?					
Konnte der Proband Ihrer Meinung nach das Tablet bedienen?					
Wie war die Bedienung der Anwendung für die Videotherapie?					
Waren alle relevanten Bereiche im Bildausschnitt für Sie erkennbar?					

Therapieabbruch: nein/ ja, wegen:___________________________________

Zusätzliche Kommentare:

Bitte geben Sie uns abschließend eine generelle Einschätzung der Videotherapie

**Datum**	
**Wie viele Videotherapien haben Sie insgesamt durchgeführt innerhalb der Studie?**	Anzahl Therapien:<br>Abgebrochene Verordnungen:
**Welche Art der Therapie wurde durchgeführt?** (mehrere Auswahlmöglichkeiten)	Allgemeine Bewegungsübungen<br>Atemtherapie<br>Allg. Hockergymnastik<br>Balance-Übungen<br>Kräftigungsübungen<br>Sonstiges:_________________________________
**Welche Hilfsmittel wurden vom Patienten verwendet?** (mehrere Auswahlmöglichkeiten)	Stuhl mit Armlehne:<br>Stuhl ohne Armlehne:<br>Hocker<br>Stab<br>Theraband<br>Wasserflasche<br>Hantel<br>Handtuch<br>Sonstiges:________________________________

	**1 (sehr gut)**	**2 (gut)**	**3 (teils gut/teils schlecht)**	**4 (schlecht)**	**5 (sehr schlecht)**
Wie war die Erfahrung mit der Videotherapie?					
Wie war die Video-Qualität?					
Wie konnten Sie ihre Patienten verstehen?					
Wie gut konnten die Patienten Sie verstehen?					
Wie konnten die Patienten Ihren Anweisungen folgen?					
Wie konnten die Patienten Ihrer Meinung nach das Tablet bedienen?					
Wie konnten Sie Ihren PC/Laptop während der Behandlung bedienen?					

	**Ja**	**Nein**
Hatten Sie Angst, dass die Person während der Therapie stürzt?		
Ist die Videotherapie für Sie eine Alternative zur normalen Physiotherapie während der Corona-Krise?		
Ist die Videotherapie für Sie eine Alternative zur normalen Physiotherapie?		
Hatten Sie das Gefühl, dass der Aufwand größer als der Nutzen ist?		
**Zusätzliche Kommentare:**		

Feedback-Interview ConVideo Physiotherapeut*innen

1. Wäre es aus Ihrer Sicht sinnvoll, Videotherapie in den Leistungskatalog aufzunehmen? Welche Vor- oder Nachteile sehen Sie?

2. Nach Ihren bisherigen Erfahrungen: Sehen Sie Unterstützungsbedarf bei der Umsetzung der Videotherapie?

Ich sehe Unterstützungsbedarf bei: (Mehrfachantworten möglich)

- ☐ Mir selbst
- ☐ Meinen Kolleg*innen / Mitarbeiter*innen
- ☐ in anderen Praxen
- ☐ kein Unterstützungsbedarf bei den oben genannten
- ☐ Sonstige:________________________________

Wobei sehen Sie Unterstützungsbedarf? (Mehrfachantworten möglich)

- ☐ PC-Kompetenz
- ☐ Software-Anwendung für die Videotherapie
- ☐ inhaltliche Aspekte bei der Durchführung einer Videotherapie
- ☐ Sonstige: _______________________________

Bitte beschreiben Sie kurz, welche konkreten Maßnahmen Sie sich vorstellen könnten:

3. Wie schätzen Sie die generelle Bereitschaft Ihrer Patient*innen zur Teilnahme an der Videotherapie ein? Was könnte Patient*innen davon abhalten, Videotherapie zu nutzen?
