# Supplementary material for: Feasibility and acceptance of video-based physiotherapy: New medical care provision for older people during the COVID-19 pandemic
Source: Z Gerontol Geriatr. 2021 Apr 30;54(4):346–52. [Article in German] doi: 10.1007/s00391-021-01899-3 (PMC8090529; doi:10.1007/s00391-021-01899-3)
Supplement: Supplementary file 3 [file 391_2021_1899_MOESM3_ESM.pdf]

Kalorienverbrauch der Freizeitaktivitäten (adaptierte Version des Minnesota Leisure Time Physical Activity Questionnaire)

Freizeitaktivität	Intensitäts-Code (I)	Gesamtdauer D [min]	I x D [kcal]
Spazierengehen	3,5		
Rasen mähen	6,0		
Gartenarbeit	4,5		
Wandern	6,0		
Fahrradfahren (draußen)	4,0		
Fahrradfahren (Ergometer)	4,0		
Tanzen	5,5		
Joggen/Walking	6,0		
Schwimmen	6,0		
Segeln	3,0		
Tennis	8,0		
Badminton	7,0		
Golf zu Fuß, ziehend	5,0		
Sonstiges: ______________			
**Summe Energieverbrauch I x D**			

Energieverbrauch pro Woche: ∑ (I x D) / 2: _____________ kcal/Woche
