# Supplementary material for: Feasibility and acceptance of video-based physiotherapy: New medical care provision for older people during the COVID-19 pandemic
Source: Z Gerontol Geriatr. 2021 Apr 30;54(4):346–52. [Article in German] doi: 10.1007/s00391-021-01899-3 (PMC8090529; doi:10.1007/s00391-021-01899-3)
Supplement: Supplementary file 4 [file 391_2021_1899_MOESM4_ESM.docx]

| TN | Alter, Geschlecht | LUCAS t0 | LUCAS t1 | LUCAS t2 | Taylor t0 | Taylor t1 | Taylor t2 | Beitragende Faktoren |
| --- | --- | --- | --- | --- | --- | --- | --- | --- |
| 1 | 78 m | frail | prefrail | robust | 751 | 4065 | 3743 | Herzschrittmacher erhalten  (3 Wochen vor Beginn der Studie) |
| 2 | 71 m | frail | prefrail | prefrail | 266 | 114 | 342 | Wegfall der Physiotherapie durch Angst vor Ansteckung (t0 zu t1) (vgl.Abb.1) |
| 3 | 67 w | robust | robust | robust | 3108 | 240 | 1471 | Akute Schmerzen des Bewegungsapparates, Muskelschwäche (t1), Anmeldung Fitness-Studio (t2) |
| 4 | 71 m | robust | robust | robust | 2110 | 2110 | 1115 | LSS, Gehstrecke stark abhängig von der Medikamentenwirkung |
| 5 | 73 w | robust | prefrail | - | 401 | 240 | - | Stärkere Schmerzen, Lungensport (COPD) fand durch Corona nicht mehr statt (t1) |
| 6 | 76 w | frail | prefrail | prefrail | 2400 | 4380 | 2168 | Akuter Schwindel,  aktive Gartenarbeit (t1) |
| 7 | 73 w | robust | frail | frail | 3294 | 1533 | 1676 | Hohes Sturzrisiko, Aktivität durch Angst vor Ansteckung reduziert (vgl. Abb. 2) |
| 8 | 64 w | post robust | prefrail | robust | 1866 | 1392 | 1521 | Hüft-Endoprothese (vor t0), Beweglichkeit besser (ab t1 keine Gehhilfen mehr) |
| 9 | 82 w | robust | robust | robust | 2246 | 1686 | 1816 | Schmerzhafte Gonarthrose, hält sich seit Corona weniger draußen auf zum Spazierengehen |
| (t0=retrospektive Befragung beim ersten Telefoninterview zur Situation vor den Corona bedingten Einschränkungen, t1= Befragung beim ersten Telefoninterview zur Situation während der Einschränkungen, t2= 6 Wochen nach dem ersten Telefoninterview; LUCAS Kategorien (von schlecht bis gut): frail, prefrail, post robust, robust [3]; MLTPAQ: durchschnittlicher Kalorienverbrauch in kcal/Woche über die vorherigen 2 Wochen [14]. Abkürzungen: TN (Teilnehmer), m (männlich), w (weiblich), LSS (Lumbale Spinalkanalstenose), COPD (Chronic Obstructive Pulmonary Disease) | | | | | | | | |

Tabelle 1: Patientencharakteristika und Assessments
