# Supplementary material for: Feasibility and acceptance of video-based physiotherapy: New medical care provision for older people during the COVID-19 pandemic
Source: Z Gerontol Geriatr. 2021 Apr 30;54(4):346–52. [Article in German] doi: 10.1007/s00391-021-01899-3 (PMC8090529; doi:10.1007/s00391-021-01899-3)
Supplement: Supplementary file 5 [file 391_2021_1899_MOESM5_ESM.docx]

| **Vorteile** | **Nachteile** |
| --- | --- |
| - als Alternative zur Sicherstellung der Patientenversorgung während Infektionswellen (3/5) | - Abschätzen der Patientensicherheit teilweise schwierig (2/5) |
| - Versorgung von Patienten, die nicht in die Praxis kommen können (medizinisch bedingte Isolation, Mobilitäts-einschränkungen, lange Anfahrtswege) (3/5) | - Patienten und teilweise auch Physiotherapeuten brauchen technische Unterstützung oder Schulung der technischen Fähigkeiten (2/5) |
| - Versorgung von Hausbesuchspatienten (Wegfall des Anfahrtswegs = Zeitersparnis, Möglichkeit zur Annahme von Patienten, die weiter entfernt wohnen) (3/5) - zunehmender personeller Knappheit an Physiotherapeuten begegnen (1/5) | - stark eingeschränkte Patienten, die keine aktiven Übungen ausführen können, können nicht versorgt werden (1/5) |
| - gut für Therapieinhalte wie:   aktive Bewegungstherapien und aktive Atemtherapie geeignet (2/5)  Edukation, Anamnese und Besprechungen mit Patienten (1/5) | - Aufgrund fehlender Hands-on Techniken ist VT kein Ersatz, sondern nur Ergänzung (4/5) |

Tabelle 2: Durch die Physiotherapeuten beschriebene Vor- und Nachteile der Videotherapie
